# Self‐Regulated Learning and Perceived Clinical Stressors in Undergraduate Nursing Students: A Cross‐Sectional Study

**DOI:** 10.1002/nop2.70745

**Published:** 2026-08-10

**Authors:** Yusuf Ölker, Aybike Olgun, Beyza Büşra Altunbaş, Esma Özşaker

**Affiliations:** ^1^ Faculty of Nursing Ege University Izmir Türkiye; ^2^ Department of Surgical Nursing, Faculty of Nursing Ege University Izmir Türkiye

**Keywords:** clinical education, clinical stress, nursing students, self‐regulated learning, undergraduate education

## Abstract

**Aim:**

To examine the relationship between self‐regulated learning and perceived clinical stressors among undergraduate nursing students engaged in clinical education.

**Design:**

A descriptive and correlational cross‐sectional study.

**Methods:**

The study was conducted with 223 undergraduate nursing students enrolled at a public university during the 2022–2023 academic year. Participants were recruited using convenience sampling. Data were collected using the Self‐Regulated Learning Scale for Clinical Nursing Practices and the Nursing Students' Perceptions of Clinical Stressors Scale. Data were analysed using descriptive statistics, non‐parametric tests, Spearman's correlation, and multiple linear regression analysis.

**Results:**

Nursing students reported relatively high levels of both self‐regulated learning and perceived clinical stressors. Students who had voluntarily chosen the nursing profession demonstrated higher self‐regulated learning scores than those who had not. Perceived clinical stressor scores differed according to gender, academic year and perceived academic achievement. A weak positive association was identified between self‐regulated learning and perceived clinical stressors. However, after adjustment for gender, academic year and academic achievement in the multiple linear regression analysis, self‐regulated learning was not an independent predictor of perceived clinical stress. These findings suggest that self‐regulated learning and clinical stress represent interconnected aspects of nursing students' clinical learning experiences, although self‐regulated learning was not an independent predictor of perceived clinical stress after adjustment for potential confounding variables.

**Impact:**

- What problem did the study address?
  ○ Limited evidence exists regarding the relationship between self‐regulated learning and perceived clinical stressors among undergraduate nursing students in clinical education settings.
- What were the main findings?
  ○ Nursing students reported high levels of both self‐regulated learning and perceived clinical stressors, and a weak positive association was observed between these constructs.
- Where and on whom will the research have an impact?
  ○ The findings may inform nurse educators, curriculum planners and academic institutions in designing educational approaches that simultaneously support learning regulation and student well‐being during clinical training.

**Reporting Method:**

STROBE checklist for cross‐sectional studies.

**Patient or Public Contribution:**

No Patient or Public Contribution. Nursing students participated only as study respondents and were not involved in the design, conduct, reporting or dissemination of the study.

## 1 Introduction

Clinical education is a fundamental component of undergraduate nursing programmes, enabling students to integrate theoretical knowledge with patient care while developing clinical competence and professional identity. Despite its essential role in preparing students for professional practice, clinical education is consistently reported as one of the most stressful aspects of nursing education (Rafati et al. 2021; Yeşil Bayülgen and Akdeniz Uysal 2022; Aydın et al. 2022). During clinical placements, nursing students encounter a variety of stressors, including fear of making mistakes, perceived inadequacy in knowledge and skills, challenging interactions with instructors, exposure to patient suffering and death, interprofessional tensions, and suboptimal clinical learning environments (Akansel et al. 2022; Ching et al. 2020; George et al. 2020; Mazalová et al. 2022; Rafati et al. 2017; Zhu et al. 2019). Prolonged exposure to such stressors may adversely affect students' self‐confidence, academic performance, emotional well‐being, and commitment to the profession (Aloufi et al. 2021; Hwang et al. 2021; Masumbuko et al. 2021). Understanding students' experiences of clinical stress is therefore important for supporting effective learning and professional development during nursing education (Weerasekara et al. 2023).

Self‐regulated learning (SRL) is recognised as a key competency in nursing education and refers to learners' ability to plan, monitor and regulate cognitive, motivational and behavioural processes in pursuit of learning goals (Dığın and İşcan Ataşen 2021; Kurt and Eskimez 2022). In clinical settings, SRL enables students to take an active role in their learning by monitoring performance, seeking feedback, adapting learning strategies and reflecting on clinical experiences. In contemporary healthcare systems characterised by rapid change and increasing performance expectations, nursing students are expected to assume greater responsibility for their own learning and professional development (Şenol and Orgun 2018; Dogu et al. 2022). These self‐regulatory skills are increasingly important for managing complex clinical learning experiences (Chen et al. 2019; Subas and Karaçay 2023).

Although SRL is frequently conceptualised as a protective factor that enhances coping and reduces academic stress, much of the existing empirical evidence derives from general academic contexts rather than authentic clinical learning environments (Phillips et al. 2015; Park et al. 2022; Rascon‐Hernan et al. 2019). In nursing education, SRL becomes particularly important during clinical placements, where students must simultaneously integrate theoretical knowledge, clinical reasoning, and professional behaviours while responding to rapidly changing patient care situations. These environments require students not only to acquire knowledge but also to continuously regulate their learning processes in response to complex situational demands. Consequently, the relationship between SRL and perceptions of clinical stress may differ from that observed in traditional academic settings.

Within nursing education research, clinical stressors and self‐regulated learning have generally been examined as separate constructs (Aydın et al. 2022; Axelsson et al. 2019; Kurt and Eskimez 2022). However, the direction and nature of the relationship between these constructs remain theoretically unclear. Limited evidence is available regarding how these variables are related within authentic clinical learning environments. Students who actively engage in self‐regulated learning may perceive clinical demands differently because of increased self‐monitoring, reflection and awareness of performance expectations. Moreover, differences across academic levels may influence both stress perception and learning regulation; yet comparative evidence across undergraduate cohorts remains limited (Admi et al. 2018; Shnaider and Warshawski 2024).

Examining the relationship self‐regulated learning and perceived clinical stressors may contribute to a better understanding of nursing students' learning experiences during clinical education. Therefore, this study aimed to determine undergraduate nursing students' levels of self‐regulated learning and perceived clinical stressors and examine the association between these constructs across undergraduate levels.

The study addressed the following research questions:

What are the levels of self‐regulated learning in clinical nursing practice among nursing students?

What are nursing students' perceptions of clinical stressors during clinical education?

Is there an association between nursing students' self‐regulated learning in clinical nursing practice and their perceptions of clinical stressors?

## 2 Methods

### 2.1 Study Design and Setting

This descriptive, correlational, cross‐sectional study was conducted during the 2022–2023 academic year among undergraduate nursing students enrolled in the Faculty of Nursing at a public university in western Turkey. The four‐year undergraduate programme includes structured clinical placements beginning in the second year in medical, surgical, paediatric, obstetrics and gynaecology, psychiatric, and community health settings under faculty supervision. The target population comprised second‐, third‐, and fourth‐year nursing students who had commenced clinical education. First‐year students were excluded because they had not yet participated in clinical placements. A non‐probability convenience sampling approach was used. All eligible students who met the inclusion criteria and were present during the data collection period were invited to participate. All stages of the study were conducted in accordance with the STROBE reporting guidelines for cross‐sectional studies, and the completed STROBE checklist is provided as Supporting Information.

### 2.2 Sample Size and Participants

Sample size estimation was based on findings reported by Dogu et al. (2022). An a priori power analysis was conducted using G*Power 3.1 for correlation analysis. Assuming a small effect size (*r* = 0.23), an alpha level of 0.05, and a statistical power of 0.95, the minimum required sample size was calculated as 197 participants.

**FIGURE 1:**
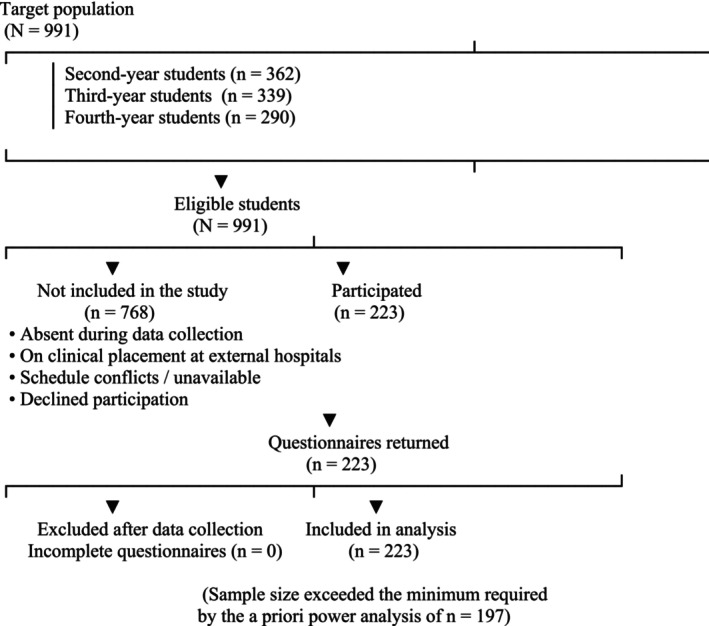
Flow diagram of participant recruitment and inclusion.

The target population comprised 991 undergraduate nursing students enrolled in the second (*n* = 362), third (*n* = 339), and fourth (*n* = 290) years of the nursing programme during the 2022–2023 academic year. The participant recruitment process is presented in Figure 1.

To account for potential incomplete responses and missing data, recruitment continued until the end of the data collection period. A total of 223 nursing students who met the inclusion criteria and provided informed consent were included in the study.

### 2.3 Inclusion Criteria

Participants were required to have completed at least one clinical placement and to provide voluntary informed consent. Students who were absent during the data collection period or who declined participation were not included. All returned questionnaires were complete; therefore, no questionnaires were excluded from the analysis due to missing data.

### 2.4 Data Collection Instruments

Data were collected using a Student Information Form, the Self‐Regulated Learning Scale for Clinical Nursing Practices (SRLS‐CNP), and the Nursing Students' Perceptions of Clinical Stressors Scale (NSPCSS‐TR). All instruments have established validity and reliability for Turkish nursing students.

### 2.5 Student Information Form

This researcher‐developed form included items on age, gender, year of study, voluntary choice of the nursing profession, and perceived academic performance.

### 2.6 Self‐Regulated Learning Scale for Clinical Nursing Practices (SRLS‐CNP)

The SRLS‐CNP was developed by Iyama and Maeda (2018), and its Turkish validity and reliability were established by Şenol and Orgun (2018). The scale consists of 16 items across two subdimensions: motivation (7 items) and learning strategies (9 items). Items are rated on a five‐point Likert scale (1 = strongly disagree to 5 = strongly agree), with total scores ranging from 16 to 80. Higher scores indicate greater use of self‐regulated learning strategies.

The original Cronbach's alpha coefficient was 0.85 (Şenol and Orgun 2018). In the present study, internal consistency was high (*α* = 0.88), with subscale coefficients of 0.86 for motivation and 0.84 for learning strategies.

### 2.7 Nursing Students' Perceptions of Clinical Stressors Scale (NSPCSS‐TR)

The NSPCSS was developed by Rafati et al. (2021), and its Turkish validity and reliability were confirmed by Aydın et al. (2022). The scale includes 22 items across six subdimensions: inappropriate clinical environment situations, instructor's inappropriate conduct, inadequate knowledge and skills, concerns related to nursing care, instructor's academic performance, and instructor's attitude towards education. Items are rated on a five‐point Likert scale (1 = never to 5 = always), with total scores ranging from 22 to 110. Higher scores indicate greater perceived clinical stress. Previous studies reported Cronbach's alpha coefficients of 0.91 (Rafati et al. 2021; Aydın et al. 2022). In the present study, internal consistency was excellent (*α* = 0.94), with subscale coefficients ranging from 0.78 to 0.89.

### 2.8 Data Collection Procedure

Students were informed about the purpose of the study and provided written informed consent prior to participation. Data were collected between May and June 2023 during students' scheduled clinical placement periods. Because nursing students were undertaking clinical placements across multiple hospitals and clinical units on different schedules, data collection was conducted throughout the academic semester to maximise participation and minimise potential selection and non‐response bias. Eligible students who were available during the data collection period were invited to participate. Questionnaires were administered anonymously in classroom settings during students' free time to minimise disruption to academic activities. Completion of the questionnaires required approximately 10–15 min. Participation was voluntary, and no incentives were provided, and completed questionnaires were collected immediately after completion to minimise missing data. These procedures were intended to reduce selection, response, and social desirability bias. Although the final sample represented only a proportion of the target population, the achieved sample size (*n* = 223) exceeded the minimum sample size required by the a priori power analysis (*n* = 197), supporting the statistical adequacy of the study.

### 2.9 Data Analysis

Data were analysed using IBM SPSS Statistics for Windows, version 25.0 (IBM Corp., Armonk, NY, USA). Descriptive statistics were presented as mean and standard deviation for normally distributed variables, median (interquartile range) for non‐normally distributed variables, and frequency and percentage for categorical variables. Normality was assessed using the Kolmogorov–Smirnov test and skewness–kurtosis values. Independent samples *t*‐tests or Mann–Whitney *U* tests were used for two‐group comparisons as appropriate. One‐way ANOVA or Kruskal–Wallis tests (followed by Dunn–Bonferroni post hoc comparisons when applicable) were applied for comparisons involving more than two groups. Associations between continuous variables were evaluated using Spearman's rank‐order correlation coefficient (Spearman's rho). Internal consistency was assessed using Cronbach's alpha. To determine whether self‐regulated learning independently predicted perceived clinical stress after controlling for potential confounding variables (gender, academic year, and academic achievement), a multiple linear regression analysis was performed. Model assumptions, including independence of residuals, were evaluated using the Durbin–Watson statistic. Statistical significance was set at *p* < 0.05.

### 2.10 Ethics Statement

Ethical approval was obtained from the Scientific Research and Publication Ethics Committee of a public University, Turkey (Approval No: 1714; Date: 1 December 2022). Written informed consent was obtained from all participants. Permission to use the scales was granted by the original authors. The study was conducted in accordance with the Declaration of Helsinki. Participants were informed that participation was voluntary, that they could withdraw from the study at any time without penalty, and that all responses would be treated confidentially and analysed anonymously.

## 3 Results

The mean age of the participants was 22.02 ± 1.84 years (range 19–36), and 85.7% were female. Nearly half of the students were in the second year (47.5%). Most participants reported choosing the nursing profession voluntarily (66.8%), and 73.5% perceived their academic performance as moderate (Table 1).

**TABLE 1:** Sociodemographic and academic characteristics of nursing students (*N* = 223).

Variables	*n*	%
Age (mean ± SD)	22.02 ± 1.84 (Min–Max: 19–36)	
Gender		
Female	191	85.7
Male	32	14.3
Academic level		
2nd year	106	47.5
3rd year	53	23.8
4th year	64	28.7
Voluntary choice of the nursing profession		
Yes	149	66.8
No	74	33.2
Perceived academic achievement		
Low	16	7.2
Moderate	164	73.5
High	43	19.3

Abbreviations: Max, maximum; Mean, arithmetic mean; Min, minimum; SD, standard deviation.

The mean total score of the SRLS‐CNP was 62.01 ± 8.45 (range 16–80). The mean motivation subscale score was 26.33 ± 4.49, including intrinsic motivation (15.47 ± 2.86) and achievement motivation (10.85 ± 2.48). The mean learning strategies subscale score was 35.67 ± 4.92, including synthesised knowledge and nursing skills (19.34 ± 2.82), multidimensional thinking (7.99 ± 1.39), and effort control (8.34 ± 1.34). Overall, the findings indicated relatively high levels of self‐regulated learning among the participants (Table 2).

**TABLE 2:** SRLS‐CNP and NSPCSS‐TR scores of nursing students.

Scale/Sub‐dimension	Scale score range (Min–Max)	Mean ± SD	Min–Max
*SRLS‐CNP*			
Motivation	(7–35)	26.33 **±** 4.49	7–35
Intrinsic motivation	(4–20)	15.47 ± 2.86	4–20
Achievement motivation	(3–15)	10.85 ± 2.48	3–15
Learning strategies	(9–45)	35.67 ± 4.92	9–45
Synthesised knowledge and nursing skills	(5–25)	19.34 ± 2.82	5–25
Multidimensional thinking	(2–10)	7.99 ± 1.39	2–10
Effort control	(2–10)	8.34 ± 1.34	2–10
SRLS‐CNP total score	(16–80)	62.01 ± 8.45	16–80
*NSPCSS‐TR*			
Inappropriate situations in the clinical environment	(6–30)	25.23 ± 4.27	10–30
Instructor's inappropriate conduct	(4–20)	17.02 ± 2.82	6–20
Instructor's academic performance	(3–15)	12.17 ± 2.26	5–15
Instructor's attitude towards education	(3–15)	11.26 ± 2.23	4–15
Student's inadequate knowledge and skills	(3–15)	12.64 ± 2.28	3–15
Concerns over nursing care	(3–15)	12.21 ± 2.39	3–15
NSPCSS‐TR total score	(22–110)	90.56 ± 13.13	44–110

Abbreviations: Max, maximum; Mean, arithmetic mean; Min, minimum; NSPCSS‐TR, Nursing Students' Clinical Stressor Perceptions Scale; SD, standard deviation; SRLS‐CNP, Self‐Regulated Learning Scale in Clinical Nursing Practice.

The mean total NSPCSS‐TR score was 90.56 ± 13.13 (range 22–110). The highest subdimension scores were observed for inappropriate situations in the clinical environment (25.23 ± 4.27), instructor's inappropriate conduct (17.02 ± 2.82), and inadequate knowledge and skills (12.64 ± 2.28). These findings suggest that environmental factors, instructor‐related issues, and perceived competency gaps were among the most prominent clinical stressors reported by students (Table 2).

No statistically significant differences were observed in SRLS‐CNP total scores according to gender, year of study, or perceived academic success (*p* > 0.05). However, students who had voluntarily chosen the nursing profession had significantly higher SRLS‐CNP total scores (*t* = 3.00, *p* = 0.004) and motivation subscale scores (*t* = 3.47, *p* = 0.001) compared with those who had not. No significant group differences were observed for the learning strategies subscale (Table 3).

**TABLE 3:** SRLS‐CNP scores according to sociodemographic and academic variables.

Variables	Motivation	Learning strategies	SRLS‐CNP total score
Gender	**Mean ± SD**	**Mean ± SD**	**Mean ± SD**
Female	26.34 ± 4.09	35.72 ± 4.75	62.06 ± 7.98
Male	26.28 ± 6.49	35.04 ± 5.94	61.68 ± 10.99
*p* *	0.957	0.776	0.085
Academic level	**Median (IQR)**	**Median (IQR)**	**Median (IQR)**
2nd year	27.50 (5.25)	36 (5.25)	63 (7.25)
3rd year	26.00 (4.50)	36 (4)	62 (8)
4th year	26.50 (4)	36 (3.75)	63 (5)
*p* **	0.181	0.082	0.401
Voluntary choice of the nursing profession			
Yes	27.20 ± 3.89	36.10 ± 4.30	63.30 ± 7.15
No	24.58 ± 5.10	34.82 ± 5.92	59.40 ± 10.15
*p* *	0.001	0.102	0.004
Perceived academic achievement			
Low	25 (8.25)	34.5 (4)	61.5 (13)
Moderate	27 (4)	36 (3)	62 (6)
High	28 (5)	36 (6)	64 (10)
*p* **	0.74	0.06	0.67

Abbreviations: IQR, interquartile range; *M*, median; Max, maximum value; Min, minimum value; SD, standard deviation; SRLS‐CNP, Self‐Regulated Learning Scale in Clinical Nursing Practices; X, arithmetic mean.

* Independent samples *t*‐test.

** Kruskal–Wallis test.

Perceived clinical stressors differed significantly according to gender, year of study, and perceived academic success (*p* < 0.05). Female students reported higher total NSPCSS‐TR scores than male students (*p* < 0.001). Third‐ and fourth‐year students had higher NSPCSS‐TR scores than second‐year students, particularly in the subdimension of inappropriate situations in the clinical environment (*p* = 0.002). Students reporting higher perceived academic success also had higher total NSPCSS‐TR scores (*p* = 0.046). No significant differences in perceived clinical stressors were observed according to whether students had chosen the nursing profession voluntarily (Table 4).

**TABLE 4:** NSPCSS‐TR scale scores of nursing students according to variables.

Variables	Inappropriate situations in the clinical environment	Instructor's inappropriate conduct	Instructor's academic performance	Instructor's attitude towards education	Student's inadequate knowledge and skills	Concerns over nursing care	NSPCSS‐TR total score
Gender	**Mean ± SD**	**Mean ± SD**	**Mean ± SD**	**Mean ± SD**	**Mean ± SD**	**Mean ± SD**	**Mean ± SD**
Female	25.42 ± 4.14	17.30 ± 2.63	12.42 ± 2.15	11.46 ± 2.17	12.85 ± 1.99	12.39 ± 2.30	91.87 ± 12.40
Male	24.06 ± 4.91	15.34 ± 3.34	10.68 ± 2.37	10.06 ± 2.25	11.37 ± 3.30	11.15 ± 2.66	82.68 ± 14.72
*p* *	0.094	< 0.001	< 0.001	0.001	0.019	0.007	< 0.001
Academic level	**Median (IQR)**	**Median (IQR)**	**Median (IQR)**	**Median (IQR)**	**Median (IQR)**	**Median (IQR)**	**Median (IQR)**
2nd year^a^	25 (6)	18 (5)	12 (4)	11 (3)	12 (3)	12 (4.25)	90 (18.25)
3rd year^b^	27 (6)	17 (3.5)	12 (3)	11 (2.5)	13 (3)	12 (3)	92 (14)
4th year^b^	27 (5)	17.50 (4)	12 (2)	12 (2.75)	13 (3)	12 (4)	94 (16.75)
*p* **	0.002; b > a	0.926	0.248	0.157	0.579	0.710	0.157
Voluntary choice of the nursing profession							
Yes	25.53 ± 4.28	17.19 ± 2.62	12.31 ± 2.18	11.44 ± 2.14	12.78 ± 2.03	12.42 ± 2.20	91.69 ± 12.75
No	24.63 ± 4.22	16.68 ± 3.17	11.90 ± 2.41	10.87 ± 2.38	12.36 ± 2.71	11.79 ± 2.68	88.27 ± 14.52
*p* *	0.142	0.209	0.204	0.073	0.196	0.086	0.066
Perceived academic achievement							
Low^a^	23 (4.75)	16 (3.75)	11.50 (3.5)	10 (4.75)	12 (4)	11.5 (4)	86 (18)
Moderate^b^	27 (5)	18 (4)	12 (3)	12 (2.75)	12 (3)	12 (4)	92.5 (16.75)
High^b^	26 (6)	17 (4)	12 (3)	11 (3)	13 (3)	12 (3)	89 (14)
*p* **	0.010; b > a	0.261	0.168	0.095	0.399	0.174	0.046; b > a

*Note:* Independent samples *t*‐test or Kruskal–Wallis test were applied as appropriate. Different superscript letters (a, b) indicate statistically significant differences between groups (*p* < 0.05), based on Dunn's post hoc test following the Kruskal–Wallis analysis. Post hoc comparisons were conducted using Dunn's test with Bonferroni adjustment.

Abbreviations: IQR, interquartile range; *M*, median; Max, maximum value; Min, minimum value; NSPCSS‐TR, Nursing Students' Clinical Stressor Perceptions Scale; SD, standard deviation; X, arithmetic mean.

* *t*‐test.

** Kruskal–Wallis test value.

Spearman correlation analysis demonstrated a weak positive association between SRLS‐CNP total scores and NSPCSS‐TR total scores (*ρ* = 0.235, *p* = 0.001). Positive correlations were also observed between SRLS‐CNP total scores and all NSPCSS‐TR subdimensions (*ρ* = 0.137–0.269, *p* < 0.05). The strongest associations were found for concerns over nursing care (*ρ* = 0.269) and instructor's attitude towards education (*ρ* = 0.242), although all correlations were small in magnitude (Table 5).

**TABLE 5:** Correlation between SRLS‐CNP and NSPCSS‐TR scores.

		SRLS‐CNP total score	Motivation	Learning strategies
NSPCSS‐TR total score	*ρ*	0.235**	0.143*	0.265**
	*p*	0.001	0.033	0.001
Inappropriate situations in the clinical environment	*ρ*	0.137*	0.049	0.192**
	*p*	0.041	0.469	0.004
Instructor's inappropriate conduct	*ρ*	0.236**	0.181**	0.236**
	*p*	0.001	0.007	0.001
Instructor's academic performance	*ρ*	0.168*	0.103	0.205**
	*p*	0.012	0.123	0.002
Instructor's attitude towards education	*ρ*	0.242**	0.114	0.300**
	*p*	0.001	0.088	0.001
Student's inadequate knowledge and skills	*ρ*	0.151*	0.114	0.148*
	*p*	0.024	0.091	0.027
Concerns over nursing care	*ρ*	0.269**	0.184**	0.288**
	*p*	0.001	0.006	0.001

Abbreviations: *ρ*, Spearman's rank‐order correlation coefficient; NSPCSS‐TR, Nursing Students' Clinical Stressor Perceptions Scale; SRLS‐CNP, Self‐Regulated Learning Scale in Clinical Nursing Practices.

* Correlation is significant at the 0.05 level (two‐tailed).

** Correlation is significant at the 0.01 level (two‐tailed).

To examine whether self‐regulated learning independently predicted perceived clinical stress after controlling for potential confounding variables, a multiple linear regression analysis was conducted (Table 6). The overall regression model was statistically significant (*F*(6,216) = 4.662, *p* < 0.001), explaining 11.5% of the variance in perceived clinical stress (*R* ^2^ = 0.115; adjusted *R* ^2^ = 0.090). After adjustment for gender, academic year, and perceived academic achievement, self‐regulated learning was no longer a statistically significant predictor of perceived clinical stress (*β* = 0.127, *p* = 0.056). However, being a second‐year student (*β* = 0.190, *p* = 0.007), being a third‐year student (*β* = 0.197, *p* = 0.005), and reporting moderate academic achievement (*β* = 0.281, *p* = 0.017) remained independently associated with higher perceived clinical stress. The model explained a modest proportion of the variance in perceived clinical stress, suggesting that additional individual, educational, and contextual factors may also contribute to students' clinical stress experiences.

**TABLE 6:** Multiple linear regression analysis predicting perceived clinical stress (NSPCSS‐TR total score) (*N* = 223).

Predictor	*B*	SE	*β*	*t*	*p*	95% CI for *B*
Constant	16.619	2.289	—	7.259	< 0.001	12.106, 21.131
Self‐regulated learning	0.064	0.033	0.127	1.920	0.056	−0.002, 0.130
Female (ref: Male)	1.368	0.789	0.112	1.732	0.085	−0.188, 2.923
Third‐year (ref: Fourth‐year)	1.858	0.662	0.197	2.806	0.005	0.553, 3.163
Second‐year (ref: Fourth‐year)	1.904	0.694	0.190	2.743	0.007	0.536, 3.273
Moderate academic achievement (ref: Low)	2.722	1.130	0.281	2.408	0.017	0.494, 4.949
High academic achievement (ref: Low)	2.428	1.272	0.226	1.908	0.058	−0.080, 4.936

*Note:* Model statistics: *R* = 0.339; *R* ^2^ = 0.115; Adjusted *R* ^2^ = 0.090; *F*(6,216) = 4.662; *p* < 0.001; Durbin–Watson = 1.950.

## 4 Discussion

This study examined nursing students' self‐regulated learning (SRL), their perceptions of clinical stressors, and the association between these constructs. The findings indicated that students reported relatively high levels of both SRL and perceived clinical stress, and that these constructs were positively associated. These results suggest that active engagement in learning regulation and heightened perceptions of stress may coexist within demanding clinical learning environments. Although the study was conducted at a single university in Turkey, the findings may offer insights into clinical learning experiences in comparable undergraduate nursing programmes.

The mean SRLS‐CNP score indicates well‐developed self‐regulated learning skills among the participants. This finding is consistent with previous studies reporting relatively high levels of SRL among nursing students (Dığın and İşcan Ataşen 2021; Dogu et al. 2022; Subas and Karaçay 2023; Elmalı Şimşek and Aksoy 2023; Hwang et al. 2021), although moderate levels have also been reported in other contexts (Chen et al. 2019; Şenol and Orgun 2018). Variability across studies may be explained by differences in measurement tools, institutional learning environments, and educational cultures. From a theoretical perspective, SRL reflects a combination of metacognitive monitoring, motivational regulation, and behavioural control processes that enable students to manage complex learning tasks. These processes are particularly important in clinical education, where students must continuously evaluate their performance, integrate feedback, and adapt their learning strategies in response to dynamic clinical situations. Supporting the development of SRL therefore remains a key objective in undergraduate nursing education.

In the present study, SRL levels did not differ significantly according to gender, year of study, or perceived academic success. Although some studies have reported associations between SRL and academic achievement or gender (Subas and Karaçay 2023; Park et al. 2022; Kurt and Eskimez 2022; Dığın and İşcan Ataşen 2021; Dogu et al. 2022; Elmalı Şimşek and Aksoy 2023; Axelsson et al. 2019; Phillips et al. 2015; Rascon‐Hernan et al. 2019), findings across the literature remain inconsistent. The absence of differences in the present study may suggest that shared instructional structures, similar clinical expectations, and common learning environments exert a stronger influence on self‐regulatory processes than demographic characteristics. Similarly, the lack of variation across academic levels may indicate that students encounter relatively stable learning expectations throughout their clinical education. In contrast, students who had voluntarily chosen the nursing profession demonstrated significantly higher SRL levels, highlighting the importance of intrinsic motivation and professional commitment in shaping students' engagement with self‐regulated learning processes. Approximately one‐third of the participants reported that they had not chosen nursing voluntarily. Although the reasons underlying this finding were not investigated in the present study, it may reflect contextual factors such as university placement outcomes, employment considerations, or family expectations. These factors may influence students' motivation, engagement with learning, and perceptions of clinical stress and warrant further exploration in future qualitative studies.

Consistent with previous research, nursing students in this study reported relatively high levels of perceived clinical stress (Hwang et al. 2021; Li et al. 2024; Rafati et al. 2021; Dias et al. 2024; Shnaider and Warshawski 2024). The highest stress levels were associated with instructor behaviours, perceived inadequacy in knowledge and skills, and environmental constraints. These findings reinforce the central role of interpersonal interactions and evaluative dynamics in shaping stress experiences during clinical placements (Mazalová et al. 2022; Shnaider and Warshawski 2024; Admi et al. 2018). Although moderate levels of stress may increase alertness and engagement with learning tasks, sustained or excessive stress may negatively influence well‐being, learning satisfaction, and academic performance (Masumbuko et al. 2021). Strengthening supportive supervision, constructive feedback, and structured learning opportunities may therefore be essential for improving clinical learning environments and reducing unnecessary stress.

Higher stress perception among female students, senior students, and those reporting higher academic success is partially consistent with previous findings, although results across studies remain mixed (Sanchez de Miguel et al. 2019; Suen et al. 2016; Ching et al. 2020; Admi et al. 2018; Liu et al. 2022). After adjusting for potential confounding variables in the multiple linear regression model, academic year and moderate academic achievement remained significant independent predictors of clinical stress, whereas gender and self‐regulated learning were no longer statistically significant. Increased clinical responsibility and greater evaluative pressure in later stages of education may heighten students' awareness of potential errors and professional expectations (Weerasekara et al. 2023). Students who perceive themselves as academically successful may also experience stronger performance expectations and self‐imposed standards, which may contribute to elevated stress perceptions. Alternatively, students who are more academically engaged may be more attentive to clinical requirements and therefore more likely to recognise and report stressors within the clinical learning environment. These findings highlight the importance of monitoring stress experiences throughout different stages of nursing education.

A key finding of this study was the positive association between self‐regulated learning and perceived clinical stressors. Given the cross‐sectional design, this relationship should be interpreted as correlational rather than causal. Although the bivariate analysis demonstrated a statistically significant positive correlation between self‐regulated learning and clinical stress, this association was attenuated after adjustment for gender, academic year, and academic achievement in the multivariable regression model and was no longer statistically significant. This finding suggests that part of the observed relationship may be explained by shared characteristics of the students rather than an independent effect of self‐regulated learning alone. The findings nevertheless indicate that higher self‐regulatory engagement may coincide with increased awareness of clinical expectations and performance standards. Students who actively monitor, evaluate, and regulate their learning processes may be more attentive to contextual demands, potential performance gaps, and evaluative pressures within clinical environments. This interpretation is consistent with cognitive appraisal perspectives of stress, which emphasise the role of individuals' evaluations of situational demands in shaping stress experiences. However, the observed association was small in magnitude, indicating that self‐regulated learning accounts for only a limited proportion of variation in perceived clinical stressors. Furthermore, the regression model explained 11.5% of the variance in clinical stress (*R* ^2^ = 0.115), suggesting that additional personal, educational, and contextual factors contribute substantially to students' stress experiences. Because both constructs were measured simultaneously, alternative interpretations remain possible. For example, greater exposure to challenging clinical situations may stimulate students to engage more actively in self‐regulatory learning processes. Longitudinal studies are required to determine how self‐regulated learning and perceived clinical stress interact over time.

The findings suggest that educational initiatives aimed at strengthening self‐regulated learning should be accompanied by strategies that address students' experiences of clinical stress and psychological well‐being. Particular attention may be warranted for students in higher academic years, as academic year emerged as an independent predictor of perceived clinical stress in the adjusted analysis. Integrating stress‐management training, emotional regulation strategies, and structured reflective learning activities into undergraduate curricula may help students maintain high levels of learning engagement while effectively coping with the demands of clinical practice (Aloufi et al. 2021). Future longitudinal research is needed to clarify the directionality of the relationship between SRL and stress and to examine how educational interventions may influence both constructs over time.

## 5 Limitations

This study has several limitations. First, the sample was drawn from a single nursing faculty, using a non‐probability convenience sampling approach, which may limit the generalisability of the findings. Although the gender distribution reflects nursing education patterns in Turkey, the small proportion of male students may have influenced gender‐based comparisons. Despite efforts to minimise selection bias by conducting data collection throughout the academic semester across multiple clinical placement sites, some degree of selection bias cannot be completely excluded because participation was voluntary. Second, all variables were measured using self‐report instruments at a single time point, introducing the possibility of response bias, social desirability bias, and common method variance. Clinical placement characteristics (e.g., ward type or patient acuity) were not controlled and may have influenced stress perceptions. Finally, the cross‐sectional correlational design precludes causal inferences. Longitudinal and multi‐centre studies are needed to clarify the directionality and temporal dynamics of the relationship between self‐regulated learning and perceived clinical stress. Despite these limitations, the study utilised validated, context‐specific measurement tools and achieved a sample size exceeding the minimum required by the a priori power analysis, enhancing confidence in the robustness of the observed findings. Future studies should incorporate additional educational, psychological, and contextual variables to better explain variation in clinical stress perceptions.

## 6 Implications for Nurse Education

The findings suggest that self‐regulated learning and perceived clinical stress may coexist rather than function as opposing constructs in clinical education. Educational approaches that focus exclusively on strengthening self‐regulated learning may therefore not necessarily reduce students' stress perceptions. Nurse educators should consider integrating structured stress‐management strategies, emotional regulation training, and guided reflective practices alongside educational initiatives that promote metacognitive awareness and strategic learning skills. Strengthening supportive supervision, constructive feedback processes, and psychologically safe clinical learning environments may further help students balance high academic engagement with emotional well‐being. Embedding both learning regulation and stress management as complementary learning outcomes within undergraduate curricula may enhance students' preparedness for complex and demanding clinical practice environments.

## 7 Conclusions

Nursing students in this study demonstrated high levels of self‐regulated learning while simultaneously reporting high levels of perceived clinical stressors. A small but statistically significant positive association was identified between these constructs. Although a weak positive correlation was observed, self‐regulated learning was not an independent predictor of perceived clinical stress after adjustment for potential confounding variables Clinical education should therefore address learning regulation and stress management as interconnected processes. Future longitudinal and intervention‐based studies are needed to clarify causal pathways and to evaluate educational approaches that support both academic engagement and psychological resilience among nursing students.

## Author Contributions

**Esma Özşaker:** conceptualization, investigation, funding acquisition, writing – original draft, methodology, validation, visualization, writing – review and editing, software, formal analysis, project administration, supervision, resources. **Beyza Büşra Altunbaş:** conceptualization, investigation, funding acquisition, writing – original draft, methodology, validation, visualization, writing – review and editing, software, formal analysis, data curation, resources. **Yusuf Ölker:** conceptualization, investigation, funding acquisition, writing – original draft, methodology, visualization, writing – review and editing, validation, software, formal analysis, project administration, data curation, supervision, resources. **Aybike Olgun:** conceptualization, investigation, funding acquisition, writing – original draft, methodology, validation, visualization, writing – review and editing, software, formal analysis, data curation, resources.

## Funding

This study was funded by The Scientific and Technological Research Council of Türkiye (TÜBİTAK) under the 2209‐A University Students Research Projects Support Programme (Grant No. 1919B012219115).

## Conflicts of Interest

The authors declare no conflicts of interest.

## Supporting information


**Data S1:** STROBE Statement—checklist of items that should be included in reports of observational studies

## Data Availability

The data that support the findings of this study are available from the corresponding author upon reasonable request.
