# Supplementary material for: Self‐Regulated Learning and Perceived Clinical Stressors in Undergraduate Nursing Students: A Cross‐Sectional Study
Source: Nurs Open. 2026 Aug 10;13(8):e70745. doi: 10.1002/nop2.70745 (PMC13456640; doi:10.1002/nop2.70745)
Supplement: Supplementary file 1 [file NOP2-13-e70745-s001.docx]

STROBE Statement—checklist of items that should be included in reports of observational studies

|  | Item No. | Recommendation | Page  No. | Relevant text from manuscript |
| --- | --- | --- | --- | --- |
| **Title and abstract** | 1 | (*a*) Indicate the study’s design with a commonly used term in the title or the abstract | 1-2 | A Cross-Sectional Study… |
|  |  | (*b*) Provide in the abstract an informative and balanced summary of what was done and what was found | 1-2 | The abstract summarises the background, aim, cross-sectional design, sample (n=223), instruments (Self-Regulated Learning Scale for Clinical Nursing Practices; Nursing Students’ Perceptions of Clinical Stressors Scale), statistical analyses (descriptive statistics, group comparisons, Spearman’s rho), and key findings (positive correlation between SRL and perceived stressors). |
| Introduction | | | |  |
| Background/rationale | 2 | Explain the scientific background and rationale for the investigation being reported | 3-4 | The introduction outlines the role of self-regulated learning in clinical education, the stressfulness of clinical environments, and the limited empirical evidence examining their coexistence in authentic clinical contexts. |
| Objectives | 3 | State specific objectives, including any prespecified hypotheses | 4 | The study aimed to examine the association between self-regulated learning and perceived clinical stressors among undergraduate nursing students. |
| Methods | | | |  |
| Study design | 4 | Present key elements of study design early in the paper | 5 | This cross-sectional and correlational study was conducted during the 2022–2023 academic year. |
| Setting | 5 | Describe the setting, locations, and relevant dates, including periods of recruitment, exposure, follow-up, and data collection | 5 | The study was conducted at a faculty of nursing at a public university during the 2022–2023 academic year. |
| Participants | 6 | (*a*) *Cohort study*—Give the eligibility criteria, and the sources and methods of selection of participants. Describe methods of follow-up  *Case-control study*—Give the eligibility criteria, and the sources and methods of case ascertainment and control selection. Give the rationale for the choice of cases and controls  *Cross-sectional study*—Give the eligibility criteria, and the sources and methods of selection of participants | 5 | The study included 223 undergraduate nursing students. Eligibility criteria and voluntary participation procedures are described. |
|  |  | (*b*) *Cohort study*—For matched studies, give matching criteria and number of exposed and unexposed  *Case-control study*—For matched studies, give matching criteria and the number of controls per case | - | Not applicable (cross-sectional study). |
| Variables | 7 | Clearly define all outcomes, exposures, predictors, potential confounders, and effect modifiers. Give diagnostic criteria, if applicable | 6-7 | The primary variables were self-regulated learning (measured using the Self-Regulated Learning Scale for Clinical Nursing Practices) and perceived clinical stressors (measured using the Nursing Students’ Perceptions of Clinical Stressors Scale). Sociodemographic characteristics (e.g., gender, year of study, voluntary choice of nursing, perceived academic performance) were also examined. |
| Data sources/ measurement | 8* | For each variable of interest, give sources of data and details of methods of assessment (measurement). Describe comparability of assessment methods if there is more than one group | 6-7 | Data were collected using validated, context-specific scales administered via structured questionnaires. Scale scoring procedures and psychometric properties are described. |
| Bias | 9 | Describe any efforts to address potential sources of bias | 2 | Potential sources of bias include single-centre sampling, self-report measurement, and cross-sectional design, which may introduce response bias and limit causal inference. (Limitations section) |
| Study size | 10 | Explain how the study size was arrived at | 5 | The final sample consisted of 223 students and exceeded the minimum required sample size based on power analysis. |

Continued on next page

| Quantitative variables | 11 | Explain how quantitative variables were handled in the analyses. If applicable, describe which groupings were chosen and why | 5-7 | Scale scores were treated as continuous variables. Means and standard deviations were calculated. Spearman’s rho was used due to non-parametric distribution assumptions. |
| --- | --- | --- | --- | --- |
| Statistical methods | 12 | (*a*) Describe all statistical methods, including those used to control for confounding | 7 | Descriptive statistics, group comparisons, and Spearman’s rho correlation analyses were conducted (p < 0.05). |
|  |  | (*b*) Describe any methods used to examine subgroups and interactions | 8-9 | Group comparisons were performed according to gender, year of study, voluntary choice of nursing, and perceived academic performance. |
|  |  | (*c*) Explain how missing data were addressed | - | No missing data were reported (all questionnaires were complete). |
|  |  | (*d*) *Cohort study*—If applicable, explain how loss to follow-up was addressed  *Case-control study*—If applicable, explain how matching of cases and controls was addressed  *Cross-sectional study*—If applicable, describe analytical methods taking account of sampling strategy | - | Not applicable beyond cross-sectional convenience sampling. |
|  |  | (*e*) Describe any sensitivity analyses | - | No sensitivity analyses were conducted. |
| Results | | | | |
| Participants | 13* | (a) Report numbers of individuals at each stage of study—eg numbers potentially eligible, examined for eligibility, confirmed eligible, included in the study, completing follow-up, and analysed | 8-9 | A total of 223 nursing students participated in the study. |
|  |  | (b) Give reasons for non-participation at each stage |  | Voluntary participation was applied; no follow-up stage. |
|  |  | (c) Consider use of a flow diagram |  | A flow diagram was not used. |
| Descriptive data | 14* | (a) Give characteristics of study participants (eg demographic, clinical, social) and information on exposures and potential confounders | 8,18 | Participant characteristics (age, gender, academic year, voluntary choice of nursing, perceived academic performance) are presented. (Table 1). |
|  |  | (b) Indicate number of participants with missing data for each variable of interest |  | No missing data. |
|  |  | (c) *Cohort study*—Summarise follow-up time (eg, average and total amount) | - | - |
| Outcome data | 15* | *Cohort study*—Report numbers of outcome events or summary measures over time |  |  |
|  |  | *Case-control study—*Report numbers in each exposure category, or summary measures of exposure |  |  |
|  |  | *Cross-sectional study—*Report numbers of outcome events or summary measures | 8-9, 19,20,21 | Mean SRL scores and perceived clinical stressor scores are reported. Correlation coefficients are presented. (Table 2,3,4). |
| Main results | 16 | (*a*) Give unadjusted estimates and, if applicable, confounder-adjusted estimates and their precision (eg, 95% confidence interval). Make clear which confounders were adjusted for and why they were included | 8-9,22 | A positive correlation was found between SRL and perceived clinical stressors (ρ = .235, p = .001). Group differences in stress levels were identified. (Table 5). |
|  |  | (*b*) Report category boundaries when continuous variables were categorized |  | Continuous variables were not categorized. |
|  |  | (*c*) If relevant, consider translating estimates of relative risk into absolute risk for a meaningful time period |  | Not applicable. |

Continued on next page

| Other analyses | 17 | Report other analyses done—eg analyses of subgroups and interactions, and sensitivity analyses |  | No additional subgroup or sensitivity analyses were conducted. |
| --- | --- | --- | --- | --- |
| Discussion | | | | |
| Key results | 18 | Summarise key results with reference to study objectives | 9-11 | Higher levels of self-regulated learning were associated with higher perceived clinical stressors. |
| Limitations | 19 | Discuss limitations of the study, taking into account sources of potential bias or imprecision. Discuss both direction and magnitude of any potential bias | 12 | Single-centre design, self-report measures, uncontrolled clinical placement characteristics, and cross-sectional design are acknowledged. |
| Interpretation | 20 | Give a cautious overall interpretation of results considering objectives, limitations, multiplicity of analyses, results from similar studies, and other relevant evidence | 11-12 | Findings challenge the assumption that SRL uniformly reduces stress and highlight the importance of integrating stress-management strategies into nursing curricula. |
| Generalisability | 21 | Discuss the generalisability (external validity) of the study results | 12 | Generalisation is limited due to single-centre sampling. |
| Other information | |  | | |
| Funding | 22 | Give the source of funding and the role of the funders for the present study and, if applicable, for the original study on which the present article is based | Title Page | This research was supported by TÜBİTAK 2209-A… funders had no role in study design, data collection, or interpretation. |

*Give information separately for cases and controls in case-control studies and, if applicable, for exposed and unexposed groups in cohort and cross-sectional studies.

**Note:** An Explanation and Elaboration article discusses each checklist item and gives methodological background and published examples of transparent reporting. The STROBE checklist is best used in conjunction with this article (freely available on the Web sites of PLoS Medicine at http://www.plosmedicine.org/, Annals of Internal Medicine at http://www.annals.org/, and Epidemiology at http://www.epidem.com/). Information on the STROBE Initiative is available at www.strobe-statement.org.
